# Activation of proinflammatory signaling by 4-hydroxynonenal-Src adducts in aged kidneys

**DOI:** 10.18632/oncotarget.10854

**Published:** 2016-07-26

**Authors:** Eun Ji Jang, Dae Hyun Kim, Bonggi Lee, Eun Kyeong Lee, Ki Wung Chung, Kyoung Mi Moon, Min Jo Kim, Hye Jin An, Ji Won Jeong, Ye Ra Kim, Byung Pal Yu, Hae Young Chung

**Affiliations:** ^1^ Department of Pharmacy, Molecular Inflammation Research Center for Aging Intervention (MRCA), Pusan National University, Busan, Republic of Korea; ^2^ Department of Physiology, The University of Texas Health Science Center at San Antonio, San Antonio, Texas, United States of America

**Keywords:** 4-HNE, Src, aged kidney, inflammation, cell senescence, Gerotarget

## Abstract

In our previous study, reactive 4-hydroxy-2-nonenal (4-HNE) was shown to activate Src (a non-receptor tyrosine kinase) by forming an adduct on binding with a specific residue of Src, leading to the activation of proinflammatory signaling pathways in cultured cells. However, to date, the deleterious roles of 4-HNE in inflammatory signaling activation in kidneys during aging have not been explored. The purpose of the present study was to document the mechanisms by which 4-HNE induces inflammation in the kidney during aging. Initial experiments revealed that activated nuclear factor-κB (NF-κB) expression was caused by 4-HNE activation, which suppressed transcriptional activity in the aged kidney. Treatment of human umbilical vein endothelial cells with 4-HNE revealed that Src caused senescence via NF-κB activation. Furthermore, our immunohistochemistry data showed that 4-HNE-adducted Src significantly increased in aged kidney tissues. The data showed age-related upregulation of downstream signaling molecules such as mitogen activated protein kinases (MAPKs), activator protein-1 (AP-1), NF-κB, and COX-2 in a cell culture cell system.

Taken together, the results of this study show that the formation of adducts between 4-HNE and Src activates inflammatory signaling pathways in the aged kidney, contributing to age-related nephropathy.

## INTRODUCTION

The molecular inflammation hypothesis provides a molecular link between oxidative stress-induced chronic inflammation and many age-related pathologic processes involving increased lipid peroxidation during aging.

It is now well documented that lipid peroxidation products such as 4-hydroxynonenal (4-HNE), 4-hydroxyhexenal (HHE), and malondialdehyde (MDA) accumulate with age. The activity of reactive aldehydes increases with enhancement of lipid peroxidation [1, 2]. These reactive aldehydes are known to cause redox disturbances and various degenerative processes, including vascular dysfunction associated with aging [1, 3, 4].

HNE is a byproduct of n-6 fatty acid peroxidation [1, 5]; it has been identified as a potent cytotoxic agent and can accumulate to levels of up to 10 μM-5 mM both *in vivo* and *in vitro* [2, 6]. 4-HNE generated by oxidative stress is thought to lead to apoptosis [4, 7], pulmonary edema [8], and cardiovascular disorders such as atherosclerosis [9].

Although there have been several studies showing that 4-HNE is increased in aged tissue or plasma, a study on the specific relationship between 4-HNE and aging has not yet been made reported. Among several hypotheses of aging, the oxidative stress hypothesis currently offers the best mechanistic description of the aging process and of age-related chronic disease processe [10]. Recent research reports provide evidence that oxidative processes are a major factor in the activation of redox-sensitive inflammatory processes, and that they act as a bridge between the normal aging process and age-related chronic diseases [11, 12].

Inflammation represents the main driving force in the progression of a large majority of human chronic diseases, as well as aging. The data available strongly suggest that 4-HNE is a key molecule in inflammation-related cell signaling, suggesting the involvement of 4-HNE in human pathologies. Specifically, several studies have shown that 4-HNE-induced inflammation is involved in COX-2 expression. [13].

A previous study revealed that the balance of protein tyrosine kinase and protein tyrosine phosphatase is important for regulating inflammatory processes and aging [13]. Furthermore, it was found that 4-HNE-activated Src (a non-receptor tyrosine kinase), among various protein tyrosine kinases and downstream signaling, directly formed adducts with a specific residue of Src, suggesting the importance of Src in inflammatory signaling pathways [14, 15].

On the basis of these findings, the present study examined changes in 4-HNE and Src as well as downstream signaling proteins related to aging *in vivo*. As the aging model, rats at 6 and 24 months of age were used and kidneys were selected as the experimental tissue, because kidneys are among the tissues most activated by aging. The data obtained from the present study provide further molecular insights into the roles of 4-HNE and Src in age-related inflammation, leading to an accelerated aging process.

## RESULTS

### Increase in 4-HNE-adducted proteins in aged kidneys

**Figure 1:**
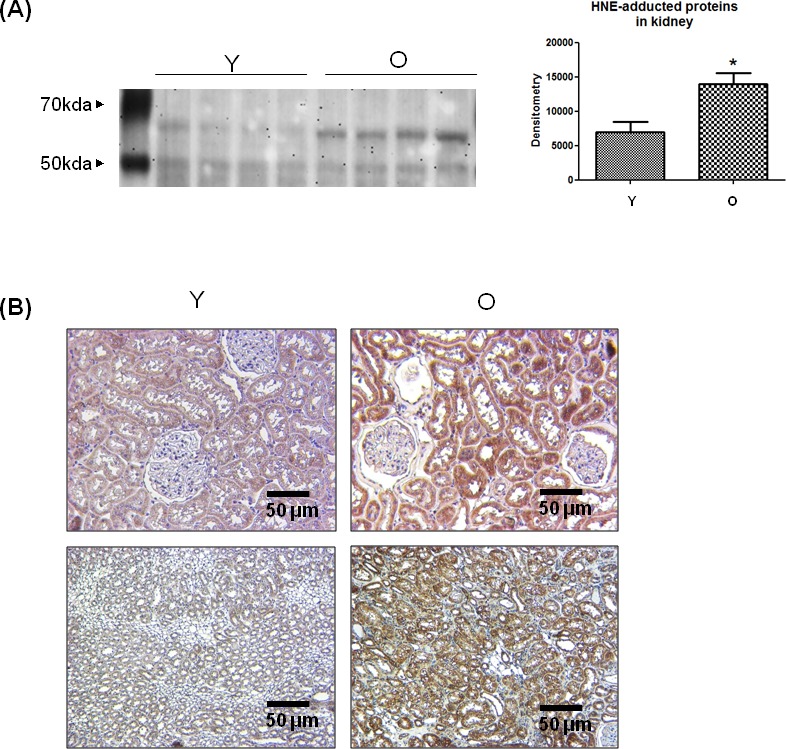
Increase in 4-HNE-adducted proteins in aged kidneys **A.** 4-HNE-adducted proteins were detected by western blot analysis using 4-HNE antibody in young and old kidney lysates. In the presentation of densitometric data, bars represent means ± SE (*n* = 4) and significance was determined using an unpaired *t* test: *p < 0.05 *vs.* young. **B.** 4-HNE was dyed brown in young and old kidney tissues in the immunohistochemistry analysis. The upper two panels represent the renal cortex and the lower two panels show the renal medulla. O, old kidney; Y, young kidney.

To confirm whether 4-HNE increases in aged kidneys, 4-HNE-adducted proteins were quantified indirectly by western blotting and immunohistochemistry using 4-HNE antibody. 4-HNE was highly adducted to proteins around 60 kD in old kidneys, and densitometric analysis showed a 2-fold increase in the levels of 4-HNE-adducted proteins (Figure 1A). Furthermore, immunohistochemistry showed that 4-HNE was strongly stained in old kidney sections (Figure 1B). These results demonstrate that 4-HNE-adducted proteins, especially 60 kD proteins, increase in aged kidneys.

### Induction of cellular senescence by 4-HNE

**Figure 2:**
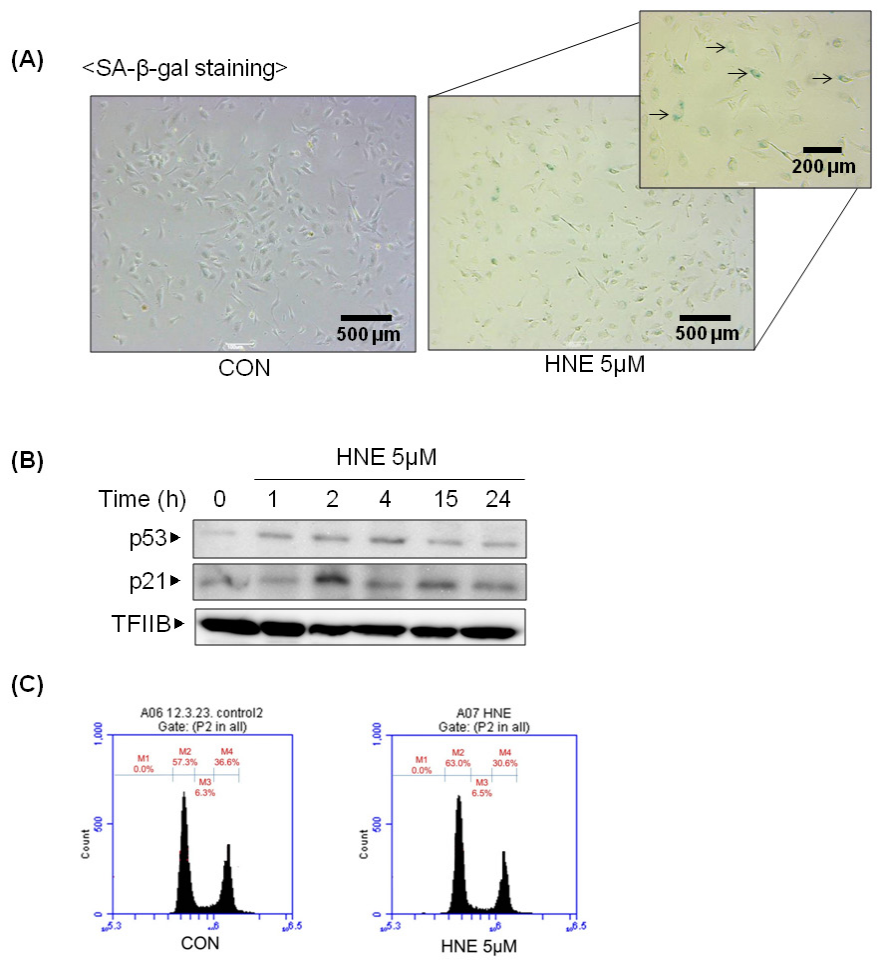
4-HNE-induced cellular senescence **A.** HUVECs were treated with 4-HNE (5 μM) for 4 h and medium was changed daily. After SA-β-gal staining, the stained cells were detected under electron microscope. The arrows indicate representative cells that are stained blue. **B.** HUVECs were treated with 5 μM 4-HNE for 1, 2, and 4 h, and after a medium change the cells were incubated for 15 and 24 h. p53 and p21 were detected by western blotting using nuclear extracts of the cells, with TFIIB used as a loading control for the nuclear extracts. **C.** The cell cycle was examined by fluorescence-activated cell sorting (FACS) with a specialized type of flow cytometry using HUVECs prepared by SA-β-gal staining. M2, G1 phase; M3, S phase; M4, G2 phase; CON, control; TFIIB, transcription factor II B.

To elucidate whether 4-HNE influences aging, 4-HNE-induced cellular senescence was investigated indirectly. Cellular senescence is a state of irreversible growth arrest. Senescent cells stop dividing but remain viable. A senescent cell phenotype includes apoptosis resistance, growth arrest, and altered gene expression, which is related to the inhibition of cell cycle proteins, such as p53, p21, pRB, and p16. SA-β-gal staining is the primary marker of cellular senescence: senescent cells are dyed blue. Figure 2A shows that cells were dyed blue when treated with 4-HNE. p21 is a cyclin-dependent kinase (CDK) inhibitor that inhibits the activity of cyclin-CDK2, -CDK1, and -CDK4/6 complexes, and thus functions as a regulator of cell cycle progression at the G1 and S phases. The expression of p21 is increased by p53. When cells were treated with 4-HNE, p21 and p53 increased and p21 was upregulated, even after a night (Figure 2B). Cells in the G1 phase were increased about 6 percent in the flow cytometric analysis, which meant that G1 arrest had partially occurred (Figure 2C). These findings suggest that 4-HNE, which increases during aging, can also inversely promote the aging process.

### Upregulation of gene expression and increased activation of Src in aged kidneys

**Figure 3:**
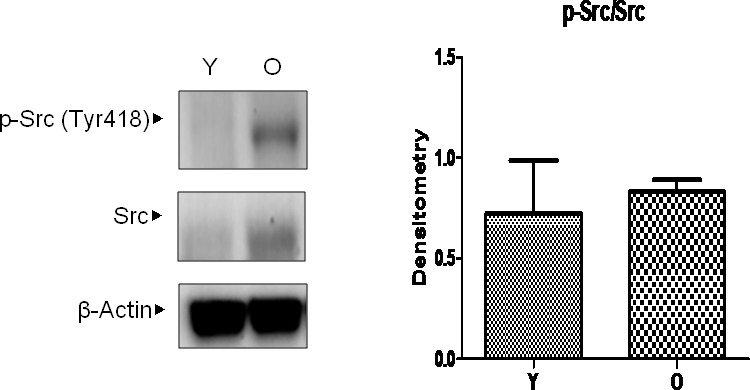
Changes in Src levels in aged kidneys Young and old kidney lysates were analyzed by western blotting. In the presentation of densitometric data, bars represent means ± SE (*n* = 6) and significance was determined using an unpaired *t* test: *p < 0.05 *vs.* young. O, old kidney; Y, young kidney.

According to previous studies, [15] Src is believed to play an important role in 4-HNE-induced inflammatory signaling as well as aging. Furthermore, because changes in Src during aging have not yet been identified, the level of Src in aged kidneys was examined. The selected binding proteins of 4-HNE in aged kidneys, total Src and active Src, which is phosphorylated on Tyr 418, were detected by western blotting analysis in young and old kidneys (Figure 3). The level of total Src was highly increased in old kidneys. However, by densitometric analysis, there was no significant difference in the ratios of p-Src and Src. These results confirm that both the activity and amount of Src, as well as 4-HNE, are increased in old kidneys.

### Binding of 4-HNE with Src in aged kidneys

**Figure 4:**
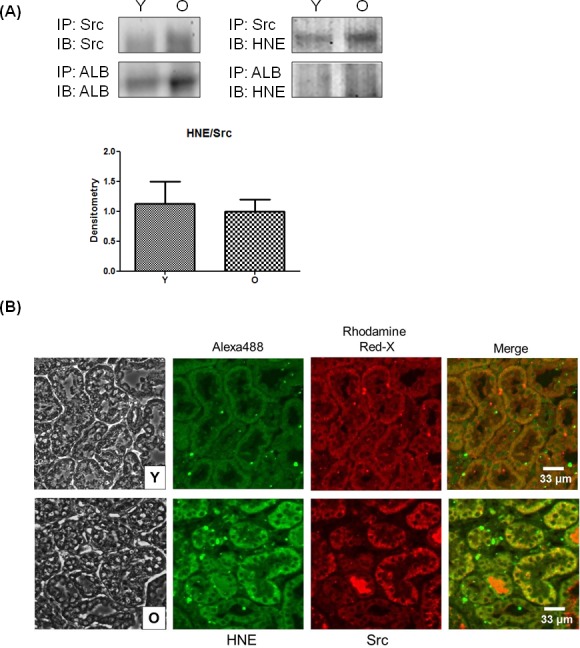
Binding of 4-HNE and Src in kidney tissues **A.** Young and old kidney lysates were immunoprecipitated with Src and albumin, and then Src, albumin, and 4-HNE were detected by western blotting. Each experiment was performed with three sets of samples. In the presentation of densitometric data, bars represent means ± SE (*n* = 3), and no statistical significance was found by unpaired *t* test. **B.** Two primary antibodies for 4-HNE and Src were used to simultaneously treat young- and old-kidney paraffin sections before double immunofluorescence staining. Alexa 488 Goat Anti-Rabbit antibody to 4-HNE shows green fluorescence and Rhodamine Red-X Goat Anti-Mouse antibody to Src shows red fluorescence. After staining, the fluorescence was observed by confocal microscopy. ALB, albumin; O, old kidney; Y, young kidney.

To confirm the interaction between Src and 4-HNE, both of which are increased during aging, an immunoprecipitation assay was performed *in vivo*. Albumin was selected as a positive control because albumin is known to form adducts with 4-HNE [16]. 4-HNE was more clearly detected in precipitates of Src than of albumin (Figure 4A). Because the level of adducted 4-HNE was increased relative to that of Src, the difference of 4-HNE/Src between young and old tissues was not seen in the densitometry analysis. Double immunofluorescence staining allowed visualization of the location and amount of 4-HNE and Src (Figure 4B). The merged picture showed that Src and 4-HNE were mostly co-localized in kidney tissue. Compared to young kidneys, the green fluorescence of 4-HNE staining was stronger in old kidneys. These findings suggest that 4-HNE and Src exist in a bound form in old kidney tissue.

### Increase in downstream signaling of Src in aged kidneys

**Figure 5:**
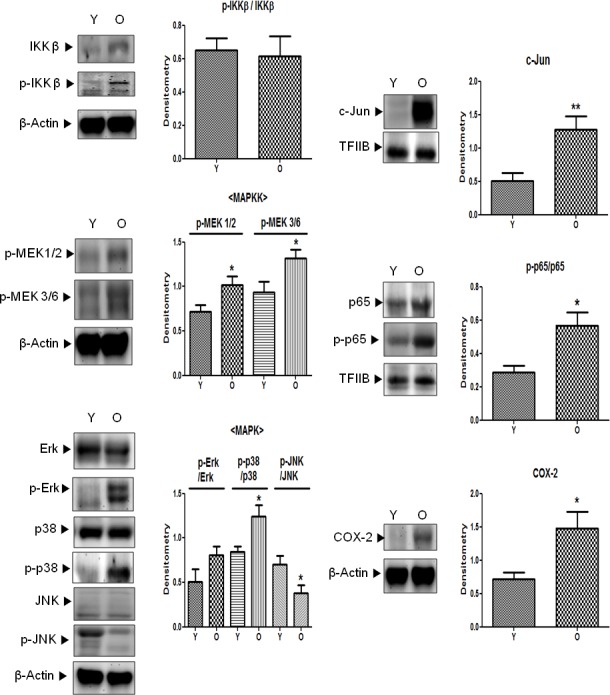
Activation of downstream signaling molecules of Src in aged kidneys p-IKKβ (Tyr199), p-MEK1/2 (Ser218/222), p-MEK3/6 (Ser189), p-Erk (Tyr204), p-p38 (Tyr182), p-JNK (Thr183/Tyr185), p-p65 (Ser536), the total form of each phosphor-form (except p-MEK), c-Jun, and COX-2 levels were analyzed by western blotting in young and old kidney lysates. In the presentation of densitometric data, bars represent means ± SE (*n* = 6) and significance was determined using an unpaired *t* test: *p < 0.05 *vs.* young. COX-2, cyclooxygenase-2; Erk, extracellular signal-regulated kinase; IKK, IκB kinase; JNK, c-Jun N-terminal kinase; MAPK, mitogen-activated protein kinase; MEK, mitogen-activated protein kinase kinase; O, old kidney; Y, young kidney.

Downstream of activated Src, there are several pathways that are related to cell motility, cell proliferation, and inflammation. Among these, MAPK signaling pathways, which are related to Src- and 4-HNE-induced inflammation, were confirmed. Contrary to the results for IKKβ, another binding molecule of Src, MAPK signaling molecules such as MEK1/2, MEK3/6, p38, and ERK were in a state of activation (phosphorylation) in old kidneys; the one exception was JNK (Figure 5A). Furthermore, COX-2, which is involved in 4-HNE-induced inflammation, was significantly increased in aged kidneys. AP-1 (c-Jun) and NF-κB (p65), the upstream transcription factors of COX-2, were also elevated in old kidneys (Figure 5B). These findings suggest that increased inflammatory signaling molecules in aged kidneys may be regulated by Src and 4-HNE.

### Effect of Src inhibition on cell senescence by 4-HNE in HUVEC cells

**Figure 6:**
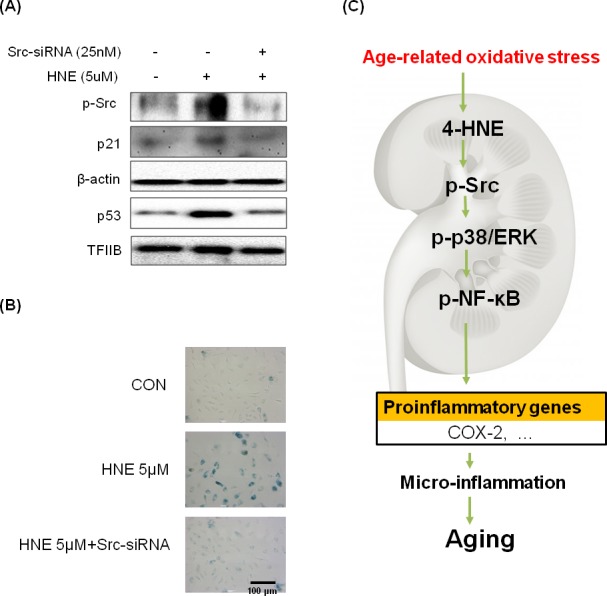
Inhibition of 4-HNE-induced cell senescence by Src-knockdown **A.** Western blot analysis was used to assess p53 and p21 protein levels in 4-HNE treatment group 48 hr after pre-treated with or without Src-siRNA cells. Samples loaded on gels were probed with β-actin and TFIIB. **B.** HUVECs (3 × 10^4^ cells) were treated with 4-HNE (5 μM) for 4 hr, 24 hr after pre-treated with Src-siRNA, and medium was changed daily. After 4 days, SA-β-gal staining was detected under electron microscope. The arrows indicate representative cells that are stained blue. **C.** Possible mechanism underlying the effect of NF-κB on 4-HNE-stimulated aging. NF-κB, nuclear transcription factor κB; COX-2, cyclooxygenase-2.

To further illustrate the importance of Src in cell senescence, we used the siRNA-mediated gene-silencing approach to Src knockdown in HUVEC cells, which was performed by treating the cells with Src-siRNA. Levels of p53 and p21 as cell senescence markers increased in 4-HNE treated groups, but were reduced by treating the cells with Src-siRNA (Figure 6A). Furthermore, we examined the SA-β-gal staining which was the primary marker of cellular senescence in Src knockdown condition. As a result, treatment with 4-HNE only showed higher dyed blue color, compared with 4-HNE treated Src-siRNA group (Figure 6B). This data indicate 4-HNE induces cellular senescence through Src signaling.

## DISCUSSION

To our knowledge, this study is the first to show that 4-HNE forms adducts with Src, thereby promoting proinflammatory NF-κB, especially in rat kidneys. In summary, we show that age-related, 4-HNE-induced Src activation causes NF-κB activation leading to senescence during the inflammatory process in aging.

4-HNE has been causally associated with various inflammatory disorders, including Alzheimer's disease [17], chronic kidney failure [18], cardiovascular disease, and atherosclerosis [19, 20]. In most of these pathologies, increased 4-HNE levels in tissue and plasma, especially 4-HNE-protein-adducted 4-HNE levels, were detected. 4-HNE-adducted protein was also found to increase in aged rat kidney tissue (Figure 1).

In a previous study of 4-HNE and its age-related changes in serum of young and old rats, the levels of 4-HNE-modified proteins were found to increase with age [21]. Many studies have demonstrated that 4-HNE or 4-HNE-modified proteins are accumulated in aged tissues. Our group reported 4-HNE-induced apoptosis in endothelial cells and aged kidneys [4]. However, whether 4-HNE directly induces aging has not been studied because of experimental limitations. Thus, cellular senescence was applied as a model. Although still debated, cellular senescence recapitulates aspects of organism aging and mimics *in vivo* aging phenotypes. The results from this study showed 4-HNE-induced cellular senescence and verified that 4-HNE can indirectly accelerate the aging process (Figure 2).

Oxidized LDL and its related lipid peroxidation products, including 4-HNE, have been reported to interact with EGF and PDGF receptors and to activate downstream signaling pathways [22, 23]. Moreover, we previously found that 4-HNE binds to Src, an NRTK, and were involved with various pathways in endothelial cells [15]. This previous work supports our findings that the stimulation of senescence in HUVEC with 4-HNE increased Src activity, and immunoprecipitation verified the direct binding between 4-HNE and Src within cells. In addition, LC-MS/MS analysis identified the specific 4-HNE binding site as Cys248 in the SH2 domain of Src. The Src knockdown experiments and dasatinib (a Src inhibitor) studies showed that reductions in Src activity inhibited p38, ERK, and AP-1 activation, as well as COX-2 expression, suggesting that Src mediates 4-HNE-induced inflammatory signaling.

Because 4-HNE is known to activate MAPK, that is, ERK, JNK, and p38, we sought to determine whether Src activation stimulates these pro-inflammatory transcriptional factors. Therefore, in the present study, it was a meaningful finding that Src increased and existed in a 4-HNE-bound form, and downstream signaling of Src, indicated by MEKs and MAPKs (ERK, p38, and JNK), that led to NF-B activation or was increased in aged kidneys shed light on the potential roles of Src in 4-HNE-induced inflammation and the aging process (Figure 5). Thus, Src could be a novel therapeutic target molecule to inhibit chronic inflammatory states, including cardiovascular disease and cancer, as well as aging.

However, because the present study investigated the role of Src in 4-HNE-induced chronic inflammation and aging, further studies are needed to identify whether Src actually plays a fundamental role in 4-HNE-induced cellular senescence and age-related disease *in vivo*. It is necessary to identify the response to 4-HNE using Src-knockout mouse.

The present study demonstrated that 4-HNE-Src adducts activate downstream inflammatory signaling pathways in aged kidney tissue. Thus, the results revealed a plausible mechanism for chronic inflammation and aging in the kidney due to the direct binding between 4-HNE and Src, as schematically depicted in Figure 6C.

## MATERIALS AND METHODS

### Animals

Young (6-month-old) and old (24-month-old) specific pathogen-free male Sprague-Dawley (SD) rats were obtained from Samtako (Osan, Korea) and housed in a controlled room (23°C ± 1°C, 55% ± 5% relative humidity, 12 h light/dark cycle) with free access to water and an ad libitum standard laboratory diet. After an acclimation period (1 week), rats at 6 (*n* = 6) and 24 (*n*= 6) months of age were sacrificed by decapitation and the kidneys were quickly removed and rinsed in ice-cold buffer [100 mM Tris, 1 mM EDTA, 0.2 mM phenylmethyl-sulfonylfluoride (PMSF), 1 μM pepstatin, 2 μM leupeptin, 80 mg/L trypsin inhibitor, 20 mM glycerophosphate, 20 mM sodium fluoride, and <2 mM sodium orthovanadate (pH 7.4)]. The tissue was immediately frozen in liquid nitrogen and stored at −80°C.

### Materials

4-HNE and p-Src (Tyr418) antibodies were purchased from Abcam (Cambridge, MA, USA) and all other antibodies used in the study were purchased from Santa Cruz Biotechnology (Santa Cruz, CA, USA) and Cell Signaling Technology (New England, Hertfordshire, UK). Polyvinylidene difluoride (PVDF) membranes were obtained from Millipore Corporation (Bedford, MA, USA). Dasatinib was purchased from LC Laboratories (Woburn, MA, USA). All other materials obtained were of the highest available grade.

### Cell culture conditions

HUVEC (human umbilical vein endothelial cells) were obtained from Lonza (Basel, Switzerland). The cells were grown in EGM™-2 (endothelial cell growth medium-2, Lonza) containing hydrocortisone, GA-1000 (gentamicin, amphotericin B), 10 ml fetal bovine serum (FBS), VEGF, hFGF-B, R3-IGF-1, ascorbic acid, and heparin. Cells were maintained at 37°C in a humidified 5% CO2 chamber with a 95% air atmosphere. Medium was replaced daily to remove non-adherent cells and cell debris. Cells were discarded after 3 months and new cells were obtained from frozen stock.

### Preparation of cytosolic and nuclear fraction from tissues

All solutions, tubes, and centrifuges were maintained at 4°C. Kidney tissues (150 mg) were homogenized with 1 ml of homogenate buffer A [10 mM HEPES (pH 7.8), 10 mM KCl, 2 mM MgCl_2_, 1 mM DTT, 0.1 mM EDTA, 0.1 mM PMSF, 1 mM pepstatin, and <1 mM p-aminobenzamidine] with a tissue homogenizer for 20 s. Homogenates were kept on ice for 15 min; then, 125 μl of 10% NP40 solution was added and mixed for 15 s. The mixture was then centrifuged at 13,000 g for 2 min, and the supernatant containing the cytosolic proteins was collected. The pelleted nuclei were washed once with 400 μl of buffer A plus 25 μl of 10% NP40, centrifuged, suspended in 50 μl of buffer C [50 mM HEPES (pH 7.8), 50 mM KCl, 300 mM NaCl, 0.1 mM EDTA, 1 mM DTT, 0.1 mM PMSF, and 10% (vol/vol) glycerol], mixed for 20 min, and centrifuged at 13,000 g for 30 min. The supernatant containing the nuclear proteins was stored at −80°C.

### Preparation of cytosolic and nuclear extracts of treated cells

Nuclear and cytosolic extracts were prepared as described previously [15]. Treated cells were washed and then scraped into 1.0 ml of ice-cold PBS and pelleted at 840 g for 5 min at 4°C. Pellets were suspended in 10 mM Tris (pH 8.0) containing 1.5 mM MgCl_2_, 1 mM DTT, 0.1% NP40, and protease inhibitors, and then incubated on ice for 15 min. Nuclei were separated from cytosols by centrifugation at 13,000 g for 15 min at 4°C. Supernatants (cytosolic fractions) were removed and pellets were suspended in 10 mM Tris (pH 8.0) containing 50 mM KCl, 100 mM NaCl, and protease inhibitors, incubated on ice for 30 min, and then centrifuged at 13,000 g for 30 min at 4°C to obtain nuclear fractions.

### Western blotting

Western blotting was carried out as described previously [24]. Homogenized samples were boiled for 5 min with gel-loading buffer (125 mM Tris-Cl, 4% SDS, 10% 2-mercaptoethanol, pH 6.8, 0.2% bromophenol blue) at a ratio of 1:1. Total protein equivalents of samples were separated by SDS-PAGE using acrylamide gels as described by Laemmli [25] and then transferred to PVDF membranes at 15 V for 1 h using a semi-dry transfer system. Membranes were then immediately placed into a blocking buffer of 10 mM Tris (pH 7.5), 100 mM NaCl, and 0.1% Tween-20 containing 1% non-fat milk. Blots were blocked at room temperature for 1 h, and then membranes were incubated with the appropriate specific primary antibody at 25°C for 1 h, followed by horseradish peroxidase-conjugated secondary antibody at 25°C for 1 h. Antibody labeling was detected using enhanced chemiluminescence, according to the manufacturer's instructions. Molecular weights were determined using pre-stained protein markers.

### Immunoprecipitation

Tissue lysates were immunoprecipitated in a buffer containing 40 mM Tris (pH 7.6), 120 mM NaCl, 5 mM EDTA, 0.1% NP40, protease inhibitors, and phosphatase inhibitors. Samples (300 μg) were precleared by incubation with a 50% slurry of protein A at 4°C for 2 h and centrifuged at 12,000 g at 4°C for 10 min. Pellets were then incubated for 3 h with the appropriate antibodies at 4°C, and incubated overnight with a 50% slurry of protein A agarose at 4°C. After washing the immunoprecipitates with buffer, immunoprecipitated proteins were analyzed by western blotting as described previously [18].

### Immunohistochemical stain

Paraffin-embedded blocks were prepared in 4-μm sections, deparaffinized, and rehydrated. After microwave pretreatment in citrate buffer (pH 6.0) for antigen retrieval, slides were immersed in 3% hydrogen peroxide for 10 minutes to block the endogenous peroxidase activity. After washing in PBS in order to block nonspecific binding, the sections were incubated with 5% skim milk for 20 min and incubated with the primary antibody, 4-HNE (1:100), which was diluted in blocking solution (Histostain-Plus kits, Invitrogen, Waltham, MA, USA) at 4°C overnight. Following incubation with the primary antibodies, the sections were treated with avidin-biotin complex reagent and DAB.

### Double immunofluorescence staining

Paraffin-embedded young and old kidney sections were deparaffinized, rehydrated, and retrieved. After blocking with 5% BSA in PBST for 30 min, sections were incubated in a mixture of two primary antibodies (rabbit polyclonal anti-4-HNE and mouse monoclonal anti-Src) overnight at 4°C. After a wash with PBS, sections were incubated with a mixture of two secondary antibodies which were raised in different species [Alexa Fluor-488 Goat Anti-Rabbit IgG (H+L) Antibody (Invitrogen) and Rhodamine Red™-X Goat Anti-Mouse IgG (H+L) Antibody (Jackson ImmunoResearch, West Grove, PA, USA)] in 1% BSA for 2 h at room temperature in the dark. Then, the sections were counterstained with 1 μg/ml of Hoechst for 5 min. After mounting, sections were stored in the dark at 4°C. Fluorescence was detected using confocal microscopy.

### Senescence-associated β-galactosidase (SA-β-gal) staining

SA-β-gal staining was performed using the Senescence-Galactosidase Staining Kit (Cell Signaling Technology, Beverly, MA, USA) according to the manufacturer's protocol. Cells were incubated at 37°C until β-gal staining became visible. Development of color was detected under a light microscope. The size of the SA-β-gal positive cell population was determined by counting up to 400 cells per dish in triplicate.

### Flow cytometric analysis

For flow cytometric analysis of the cell cycle distribution, cells were plated at 50% confluency in a 100-mm dish. After treatment with 4-HNE, cells were collected. The cells were washed in 1% BSA, fixed in chilled 95% ethanol, and stained with cold propidium iodine (PI, Sigma-Aldrich Chemicals, St. Louis, MO, USA) staining solution (10 μg/ml PI and 100 μg/ml RNase in PBS), followed by incubation in the dark for 30 min at room temperature. Data acquisition and analysis were carried out using a flow cytometry system (Accuri Cytometers, Inc., Ann Arbor, MI, USA).

### Small interfering RNA-mediated gene silencing

To knockdown Src in HUVEC cells, we utilized scrambled or Src-siRNAs obtained from a commercial source (IDT, Coralville, Iowa, USA). Transfection was carried out using the Lipofectamine 2000 reagent (Invitrogen, Grand Island, New York, USA). The cells were treated with scramble or Src siRNA-Lipofectamine complexes (25 nM) in Opti-MEM (Invitrogen) without serum. After incubation for 4 h, the transfection medium was replaced with fresh medium, and the cells were incubated for another 48 h, during which they were treated with HNE (5 μM) at the indicated times.

### Statistical analysis

One-way analysis of variance (ANOVA) was used to analyze differences among three or more groups. Differences in the means of individual groups were assessed by Bonferroni's post hoc test. Student's t test was used to analyze differences between two groups. Values of *p* < 0.05 were considered statistically significant. Analyses were performed using GraphPad Prism 5 (GraphPad software, La Jolla, CA, USA).

## ABBREVIATIONS

NF-κB: Nuclear transcription factor κB
COX-2: Cyclooxygenase-2
AP-1: Activator protein-1
4-HNE: 4-hydroxynonenal
MAPKs: Mitogen activated protein kinases

## References

[1] Esterbauer H, Schaur RJ, Zollner H (1991). Chemistry and biochemistry of 4-hydroxynonenal, malonaldehyde and related aldehydes. Free Radic Biol Med.

[2] Chiarpotto E, Biasi F, Scavazza A, Camandola S, Dianzani MU, Poli G (1995). Metabolism of 4-hydroxy-2-nonenal and aging. Bioche Biophys Res Commun.

[3] Wang DS, Iwata N, Hama E, Saido TC, Dickson DW (2003). Oxidized neprilysin in aging and Alzheimer's disease brains. Biochem Biophys Res Commun.

[4] Lee JH, Jung KJ, Kim JW, Kim HJ, Yu BP, Chung HY (2004). Suppression of apoptosis by calorie restriction in aged kidney. Exp Gerontol.

[5] Uchida K (2003). 4-Hydroxy-2-nonenal: a product and mediator of oxidative stress. Prog Lipid Res.

[6] Toyokuni S, Uchida K, Okamoto K, Hattori-Nakakuki Y, Hiai H, Stadtman ER (1994). Formation of 4-hydroxy-2-nonenal-modified proteins in the renal proximal tubules of rats treated with a renal carcinogen, ferric nitrilotriacetate. Proc Natl Acad Sci USA.

[7] Hamilton RF, Li L, Eschenbacher WL, Szweda L, Holian A (1998). Potential involvement of 4-hydroxynonenal in the response of human lung cells to ozone. Am J Physiol.

[8] Compton CN, Franko AP, Murray MT, Diebel LN, Dulchavsky SA (1998). Signaling of apoptotic lung injury by lipid hydroperoxides. J Trauma.

[9] Uchida K (2000). Role of reactive aldehyde in cardiovascular diseases. Free Radic Biol Med.

[10] Yu BP (1996). Aging and oxidative stress: modulation by dietary restriction. Free Radic Biol Med.

[11] Chung HY, Kim HJ, Kim JW, Yu BP (2001). The inflammation hypothesis of aging: molecular modulation by calorie restriction. Ann N Y Acad Sci.

[12] Chung HY, Kim HJ, Kim KW, Choi JS, Yu BP (2002). Molecular inflammation hypothesis of aging based on the anti-aging mechanism of calorie restriction. Microsc Res Tech.

[13] Kumagai T, Matsukawa N, Kaneko Y, Kusumi Y, Mitsumata M, Uchida K (2004). A lipid peroxidation-derived inflammatory mediator: identification of 4-hydroxy-2-nonenal as a potential inducer of cyclooxygenase-2 in macrophages. J Biol Chem.

[14] Jung KJ, Lee EK, Yu BP, Chung HY (2009). Significance of protein tyrosine kinase/protein tyrosine phosphatase balance in the regulation of NF-kappaB signaling in the inflammatory process and aging. Free Radic Biol Med.

[15] Jang EJ, Jeong HO, Park D, Kim DH, Choi YJ, Chung KW, Park MH, Yu BP, Chung HY (2015). Src tyrosine kinase activation by 4-Hydroxynonenal upregulates p38/AP-1 signaling and COX-2 expression in endothelial cells. Plos One.

[16] Moreau R, Heath SH, Doneanu CE, Lindsay JG, Hagen TM (2003). Age-related increase in 4-hydroxynonenal adduction to rat heart alpha-ketoglutarate dehydrogenase does not cause loss of its catalytic activity. Antioxid Redox Signal.

[17] Volkel W, Sicilia T, Pahler A, Gsell W, Tatschner T, Jellinger K, Leblhuber F, Riederer P, Lutz WK, Gotz ME (2006). Increased brain level s of 4-hydroxy-2-nonenal glutathione conjugates in severe Alzheimer's disease. Neurochemi stry International.

[17] Siems W1, Carluccio F, Grune T, Jakstadt M, Quast S, Hampl H, Sommerburg O (2002). Elevated serum concentration of cardiotoxic lipid peroxidation products in chronic renal failure in relation to severity of renal anemia. Clin Nephrol.

[19] Leonarduzzi G, Robbesyn F, Poli G (2004). Signaling kinases modulated by 4-hydroxynonenal. Free Radic Bioi Med.

[20] Uchida K (2000). Role of reactive aldehyde in cardiovascular diseases. Free Radic Bioi Med.

[21] Kim CH, Zou Y, Kim DH, Kim ND, Yu BP, Chung HY (2006). Proteomic analysis of nitrated and 4-hydroxy-2-nonenal-modified serum proteins during aging. J Gerontol A Biol Sci Med Sci.

[22] Negre-Salvayre A, Vieira O, Escargueil-Blanc I, Salvayre R (2003). Oxidized LDL and 4-hydroxynonenal modulate tyrosine kinase receptor activity. Mol Aspects Med.

[23] Zhang H, Forman HJ (2015). 4-Hydroxynonenal activates Src through a non-canonical pathway that involves EGFR/PTP1B. Free Radic Biol Med.

[24] Kim JM, Lee EK, Kim DH, Yu BP, Chung HY (2010). Kaempferol modulates pro-inflammatory NF-kappaB activation by suppressing advanced glycation endproducts-induced NADPH oxidase. Age (Dordr).

[25] Laemmli UK (1970). Cleavage of structural proteins during the assembly of the head of bacteriophage T4. Nature.

